# Protective Effects of Platelet-rich plasma for in vitro Fertilization of Rats with Ovarian Failure Induced by Cyclophosphamide

**DOI:** 10.1055/s-0041-1741451

**Published:** 2022-02-25

**Authors:** Özcan Budak, Mehmet Sühha Bostancı, Veysel Toprak, Songül Doğanay, Osman Köse

**Affiliations:** 1 Department of Histology and Embryology and Artificial Reproductive Techniques, Faculty of Medicine, Sakarya University, Sakarya, Turkey; 2 Department of Obstetrics and Gynecology and Artificial Reproductive Techniques, Faculty of Medicine, Sakarya University, Sakarya, Turkey; 3 Department of Obstetrics and Gynecology, Private Tatvan Can Hospital, Bitlis, Turkey; 4 Department of Physiology, Faculty of Medicine, Sakarya University, Sakarya, Turkey; 5 Specialist of Gynecological Oncology, Department of Obstetrics and Gynecology Faculty of Medicine, Sakarya University, Sakarya, Turkey

**Keywords:** ovary, premature ovarian insufficiency, cyclophosphamide, in vitro fertilization, platelet-rich plasma, ovário, insuficiência ovariana prematura, ciclofosfamida, fertilização in vitro, plasma rico em plaquetas

## Abstract

**Objective** Premature ovarian insufficiency (POI) contributes significantly to female infertility. Cyclophosphamide (CYC has adverse effects on folliculogenesis. Platelet-rich plasma (PRP) is an autologous product rich in many growth factors. We evaluated the protective effect of PRP on in vitro fertilization in female rats with CYC-induced ovarian damage.

**Methods** Twenty-eight adult female Sprague-Dawley rats were randomly divided into four groups. Group 1 (control-sodium chloride 0.9%; 1 mL/kg, single-dose intraperitoneal [IP] injection); group 2 (CYC), 75 mg/kg, single-dose IP injection and sodium chloride 0.9% (1 mL/kg, single-dose IP injection); group 3 CYC plus PRP, CYC (75 mg/kg, single-dose and PRP (200 μl, single-dose) IP injection); and group 4 (PRP, 200 μl, single-dose IP injection).

**Results** In the comparisons in terms of M1 and M2 oocytes, it was observed that the CYC group presented a significantly lower amount than the control, CYC/PRP, and PRP groups. (for M1, *p* = 0.000, *p* = 0.029, *p* = 0.025; for M2, *p* = 0.009, *p* = 0.004, *p* = 0.000, respectively). The number of fertilized oocytes and two-celled good quality embryos was found to be statistically significant between the CYC and control groups, CYC + PRP and PRP groups ( *p* = 0.009, *p* = 0.001, *p* = 0.000 for oocytes, respectively. For embryos; *p* = 0.016, *p* = 0.002, *p* = 0.000).

**Conclusion** Platelet-rich plasma can protect the ovarian function against damage caused by CYC, and, in addition, it improves oocyte count and the development of embryos as a result of oocyte stimulation during the IVF procedure.

## Resumo

**Objetivo** A insuficiência ovariana prematura (POI) contribui significativamente para a infertilidade feminina. A ciclofosfamida (CYC) tem efeitos adversos na foliculogênese. O plasma rico em plaquetas (PRP) é um produto autólogo rico em muitos fatores de crescimento. Avaliamos o efeito protetor do PRP na fertilização in vitro em ratas com lesão ovariana induzida por CYC.

**Métodos** Vinte e oito ratas Sprague-Dawley adultas foram divididas aleatoriamente em quatro grupos. Grupo 1 (controle - cloreto de sódio 0,9%; 1 mL/kg, injeção intraperitoneal [IP] em dose única); grupo 2 (CYC), 75 mg/kg, injeção IP de dose única e cloreto de sódio 0,9% (1 mL/kg, injeção ip de dose única); grupo 3 CYC + PRP, CYC (75 mg/kg, dose única e PRP (200 μl, dose única) injeção IP); e grupo 4 (PRP, 200 μl, injeção IP de dose única).

**Resultados** Nas comparações em termos de ovócitos M1 e M2, observou-se que o grupo CYC apresentou uma quantidade significativamente menor que os grupos controle, CYC/PRP, e PRP. (Para M1, *p* = 0,000, *p* = 0,029, *p* = 0,025; para M2, *p* = 0,009, *p* = 0,004, *p* = 0,000, respectivamente). O número de oócitos fertilizados e embriões bicelulares de boa qualidade foi considerado estatisticamente significativo entre os grupos CYC e controle, CYC + PRP e grupos PRP ( *p* = 0,009, *p* = 0,001, *p* = 0,000 para oócitos, respectivamente. Para embriões, *p* = 0,016, *p* = 0,002, *p* = 0,000).

**Conclusão** O PRP pode proteger a função ovariana contra os danos causados pelo CYC e, além disso, proporciona melhora na contagem de oócitos e no desenvolvimento de embriões como resultado da estimulação ovariana durante o procedimento de fertilização in vitro.

## Introduction

While ovarian reserve is defined as the number of follicles/oocytes present in the ovaries, premature ovarian insufficiency (POI) is defined as a decrease in ovarian functions and oocyte reserve before the age of 40. 1 The prevalence of POI is estimated to be around 1 to 3% among women when the general population is evaluated. 2 Most POIs are classified as idiopathic. 3 However, the pathophysiology of POI is thought to be related to genetic factors, radiotherapy, and chemotherapy factors, autoimmune disorders, and infections. 3 Premature ovarian insufficiency results in premature loss of ovarian function, major health problems, and infertility, especially as a result of the decreased number of oocytes in the ovaries due to accelerated atresia. In case of sufficient residual ovarian reserve, in vitro fertilization (IVF) with autologous oocytes obtained by ovarian stimulation is an effective treatment for women with POI. 4

Platelet-rich plasma (PRP) is an autologous product rich in many growth factors, such as platelet-derived growth factor (PDGF), transforming growth factor (TGF)-β, and vascular endothelial growth factor (VEGF). 5 6 Growth factors in PRP stimulate chemotaxis, proliferation, and differentiation of stem cells and angiogenesis in a way that accelerates tissue repair. 7 8 Platelet-rich plasma, which is an inexpensive product compared with many other agents, has many advantages, such as being easy to obtain and having an antimicrobial effect as well as being an autologous product. 9

Alkylating chemotherapy agents such as cyclophosphamide (CYC), which are highly gonad-toxic, cause a decrease in ovarian function and have detrimental effects on the female reproductive organs. 10 These effects of CYC are primarily due to the inhibition of DNA synthesis and function and induction of DNA damage. Cyclophosphamide has been shown to reduce primitive follicles, oocytes, and granulosa cells on eggs by inducing apoptosis, inhibiting angiogenesis, thus causing ovarian atrophy. 11

Pathological changes in CYC-generated POI patterns are similar to clinical observations in POI patients, and these pathological changes in the POI model can be reversed with drugs. 12

Growth factors such as VEGF, EGF, PDGF, and TGF-have been shown to have protective effects on ovarian damage. 6 13 14 15 Platelet-rich plasma has been found to have a predominant positive effect on ovarian cortex volume, antral follicle number and antral follicle diameter on ovarian damage caused by CYC. 12 16

There are various medical treatments, such as immunomodulating therapies, apoptotic inhibitors, antioxidant therapies, IVF, and embryo transfer using donor oocytes to restore impaired ovarian function and/or restore fertility in women with POI. 17 Women with POI require significantly higher doses of exogenous gonadotropin to initiate folliculogenesis compared with patient groups with normal ovarian reserve. 4 They commonly have a poor response to stimulation, with only four or fewer follicles available for oocyte retrieval. 4 It seems that not every approach applied to remedy this situation can be created as effective or guaranteed for successful management. 4 The protective and curative effect of PRP at the level of folliculogenesis in CYC-induced ovarian damage has been shown in previous studies. 12 16 A recent study has shown that intraovarian injection of autologous PRP has improved IVF results in women with primary ovarian insufficiency. 18 The aim of this study is to investigate the protective effect of PRP on in vitro fertilization in female rats with CYC-induced ovarian damage.

## Methods

The study was conducted in Sakarya University's SÜDETAM laboratory under the authority of Sakarya University's experimental animal ethics committee on 04/11/2020 under decision No.62. Applications for all research animals were performed according to the “The European Commission Directive 86/609/ECC guideline” protocol. Twenty-eight adult female Sprague-Dawley rats (weight 200–250 g; age 65–75 days) were provided by the Sakarya University Animal Reproduction Center and housed in groups with ad libitum food and water in the Animal Laboratory of Sakarya University. The holding room was maintained at room temperature of 22 ± 2°C with humid conditions (45–55%) and a 12-hour light/day cycle.

The rats were randomly divided into four different experimental (Exp.) groups:

Group I (control group) received sodium chloride 0.9% (1 mL/kg, single dose) intraperitoneal (IP) injection on the 1 ^st^ , 8 ^th^ , and 15 ^th^ days.

Group II (CYC group) received cyclophosphamide (CYC) (75 mg/kg, single dose) IP injection on the 1 ^st^ , 8 ^th^ , and 15 ^th^ , days.

Group III (CYC + PRP group) received CYC (75 mg/kg, single dose) and PRP (200 µl, single dose) IP injection on the 1 ^st^ , 8 ^th^ , and 15 ^th^ days.

Group IV (PRP only group) received PRP (200 µl, single dose) IP injection on the 1 ^st^ , 8 ^th^ , and 15 ^th^ days.

The stage of the estrous cycle of the rats was determined by performing daily vaginal smears after acclimation. Rats determined to have at least 3 consecutive 4-day estrous cycles were prepared for in vitro fertilization (IVF). All the rats were subjected to the IVF protocol to create hyperstimulation. On the day the stimulation was completed, female rats were sacrificed, and their oocytes were collected.

Human tubal fluid (HTF) medium (Cat. No. 90166, Irvine Scientific, Santa Ana, CA, USA) was used for sperm preincubation, fertilization, and embryo transfer. For sperm preincubation, a 200 mL droplet was used. For oocyte collection and IVF, a 100 mL volume droplet was used. Embryos were washed by passing through four such droplets. Each droplet was placed on a 35-mm culture dish (Nunc, Cat. No.63754, Denmark), covered with liquid paraffin oil (Cat. No. 9305, Irvine Scientific), and kept at 37°C under 5% CO2 in humidified air overnight.

The ovaries were stimulated through the IP route for both ovaries in the female rats. For the first injection, we used an IP injection of 150 to 300 internal units (IUs)/kg of pregnant mare serum gonadotropin (PMSG) (Chronogest/PMSG, Intervet, Istanbul, Turkey), followed ∼ 48 hour later by 150 to 300 IUs/kg of human chorionic gonadotropin (hCG; Gonatropin, Chorulon Intervet, Istanbul, Turkey). At 17 to 19 hours after hCG administration, 15 IUs of PMSG were administered. 19 All the rats were weighed and anesthetized by an intramuscular administration of 50 mg/kg ketamine hydrochloric acid (Ketalar; Eczacibasi Warner-Lambert Ilac Sanayi, Levent, Istanbul, Turkey) and 7 mg/kg xylazine hydrochloric acid (Rompun, Bayer Sisli, Istanbul, Turkey). After immobilizing the rats on a standard surgery board, blood samples were collected to measure the level of serum anti-Mullerian hormone (AMH). The aseptic technique was used to make a ventral midline incision to expose the reproductive organs, and the oviducts were removed. In this manner, the oocytes were collected from removed ovaries. To incubate the oocytes, HTF medium with the addition of 4 mg/ml of human serum albumin (HSA) was cultured for 1 day before being placed in an incubator at 37°C and 5% CO _2_ . Culture drops were prepared as group cultures on the culture dish under mineral oil. Fertilization, 2 washes, and culture drops were prepared in 500 µl, 150 µl, and 150 µl amounts, respectively. The oocytes and capacious sperm (approximate concentration 1 × 106 ml-1) were transferred to the fertilization drops. Then, fertilization was checked, and the fertilized oocytes were washed and transferred to culture drops, and the resulting embryos were monitored up to the blastocyst stage. 20

Before the oocyte collection, a mixture of 75 mg/kg of ketamine (Ketasol, Richter Pharma, Austria) and 10 mg/kg of xylazine (Basilazin, Bavet, Turkey) was applied intraperitoneally to a male rat, and then the rat was euthanized. Following the euthanasia procedure, the male reproductive system was surgically opened from the abdomen, and the left and right epididymis were separated from the testicles and transferred to HTF medium containing 1 ml of HTF (Cat. No. 90168, Irvine Scientific, USA) and 4 mg/ml of bovine serum albumin (BSA). The epididymis was carefully peeled off using forceps, and the sperm were transferred into petri dishes and incubated at 37°C for 30 minutes before in vitro fertilization. 21

Approximately 6.5 hours after insemination, the oocytes were washed 3 times with HTF medium and cultured as above. At 7 to 8 hours after insemination, the oocytes were checked for sperm penetration or pronuclear formation under an inverted microscope to identify any polyspermic fertilization or parthenogenetic embryos (∼ 6.5% of the total). After culturing for a further 20 hours, the numbers of 2-cell stage embryos were counted; these were defined as fertilized embryos.

Eight mature male Sprague-Dawley rats were used to prepare PRP. Blood samples were taken from these rats by heart puncture from the right ventricle under anesthesia and taken into test tubes containing 3.2% sodium citrate (Merck, Darmstadt, Germany) at the rate of 9/1 blood/citrate. After the blood samples were centrifuged at 400 × for 10 minutes, the upper part of the plasma containing the platelets and buffy coat was transferred to another tube and centrifuged again at 800 × g for 10 minutes. This tube contained platelet deposits and some red blood cells (an erythrocyte-platelet cluster). By removing the upper ⅔ of the supernatant containing platelet-poor plasma, the remaining layer (lower ⅓) was accepted as PRP. The final fraction, containing 2.4 × 106 platelets/ml, was ∼ 3.9 times larger than the blood platelet count (570,000 platelets/μl). We used fresh PRP per administration.

Anti-Mullerian hormone was quantitatively estimated in rat serum samples using enzyme-linked immunosorbent assay (ELISA) kits (MyBioSource, Rat AMH ELISA Kit Catalog No: MBS2509909, San Diego, California, USA).

Statistical analyses were performed using the IBM SPSS Statistics for Windows, Version 24.0 software (IBM Corp., Armonk, NY, USA). The Shapiro-Wilk test was used to evaluate the normal distribution of the data. For the comparison of more than two variables, one-way analysis of variance (ANOVA) was used for normally distributed data and the Kruskal-Wallis test was used for data that did not show normal distribution. To determine which group was different from the others, the Tukey honestly significant difference (HSD) test was used for variables with homogeneous variances, and the Tamhane T2 test for non-homogeneous variables. The results are given as mean ± standard error (SE). The statistical evaluation was considered significant when *p* < 0.05 for each test.

## Results

**Fig. 1:**
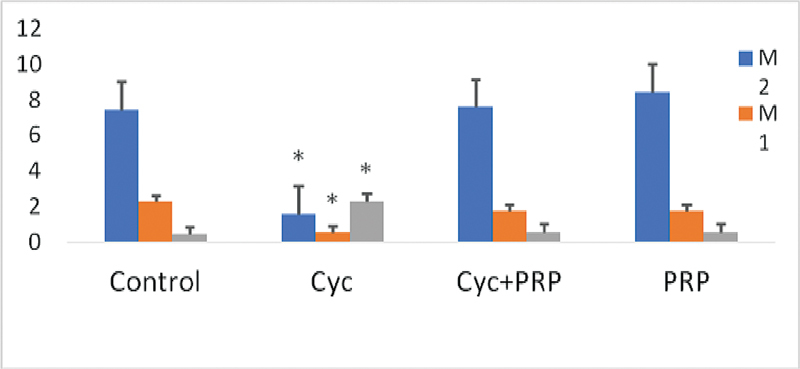
Comparison of the mean of metaphase I (M1), metaphase II (M2) oocytes and germinal vesicles (GVs) in experimental groups. Abbreviations: Control, control group; CYC, cyclophosphamide administered group; CYC + PRP, cyclophosphamide and platelet rich plasma applied group; PRP, platelet-rich plasma applied group. * *p* < 0.05 compared with the control, CYC + PRP, and PRP groups. Values are given as mean and standard error.

**Fig. 2:**
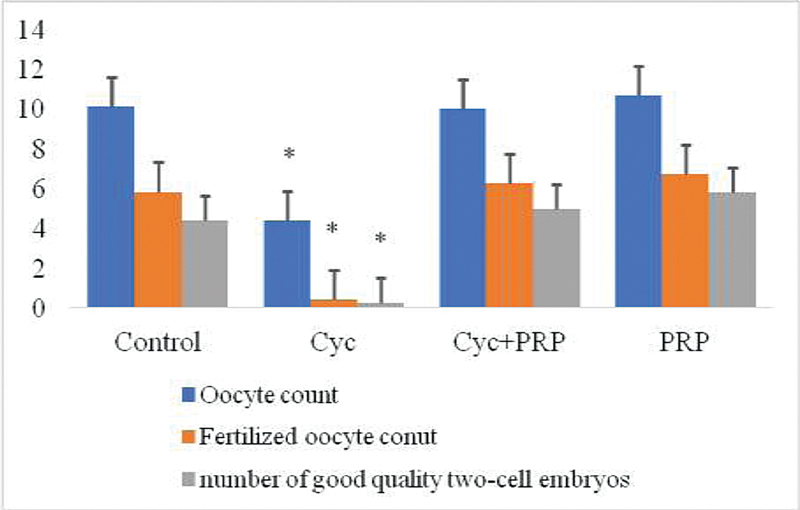
Comparison of the mean numbers of total oocytes, fertilized oocytes, and two-celled good quality embryos in the experimental groups. Abbreviations: Control, control group; CYC, cyclophosphamide administered group; CYC + PRP, cyclophosphamide and PRP applied group; PRP, platelet-rich plasma applied group. * *p* < 0.05 compared with control, CYC + PRP, and PRP group. Values are given as mean and standard error.

The oocytes were classified as germinal vesicle (GV), metaphase I (M1), and metaphase II (M2). To compare the meiotic progression during oocyte maturation in different systems, the average time that each stage of nuclear progression takes was calculated. This method was previously described by Sirard et al. 22 As a result of the statistical evaluation made in the light of this situation, it was seen that only cyclophosphamide (CYC) application decreased the average number of M1 and M2, increased the number of GVs, and PRP application prevented this effect of CYC ( Fig. 1 ). In the comparisons in terms of M1 and M2 numbers, it was observed that the CYC group presented a significantly lower number than the control, CYC/PRP, and PRP groups (for M1, respectively: *p* = 0.000, *p* = 0.029, *p* = 0.025; for M2, respectively: *p* = 0.009, *p* = 0.004, *p* = 0.000). In the evaluation made in terms of the GVs number, it was observed that the GVs number increased in the CYC group, and the PRP application decreased the GVs number. In the comparisons between groups, the GV value in the CYC group was significantly higher compared with the control, CYC + PRP, and PRP groups ( *p* = 0.001, *p* = 0.003, *p* = 0.003, respectively). When the CYC + PRP group was compared with the control and PRP groups, there was no significant difference in terms of MI, MII, GV, and oocyte count ( *p* > 0.05). The average number of oocytes, fertilized oocytes and two-celled good quality embryos belonging to the groups are presented in Fig. 2 . The mean oocyte count was statistically significantly lower in the CYC group compared with the control, CYC + PRP, and PRP groups ( *p* = 0.000 for each). When the CYC + PRP group and the control and PRP groups were compared in terms of mean oocyte count, there was no statistically significant difference between the groups ( *p* > 0.05). The mean number of fertilized oocytes and two-celled good quality embryos was the lowest in the CYC group, while it was highest in the PRP only group. In the comparison between the groups, the number of fertilized oocytes and two-celled good quality embryos was found to be statistically significant between the CYC group and control, CYC + PRP, and PRP groups ( *p* = 0.009, *p* = 0.001, *p* = 0.000 for fertilized oocytes, respectively. for the number of good quality embryos; *p* = 0.016, *p* = 0.002, *p* = 0.000).

**Fig. 3:**
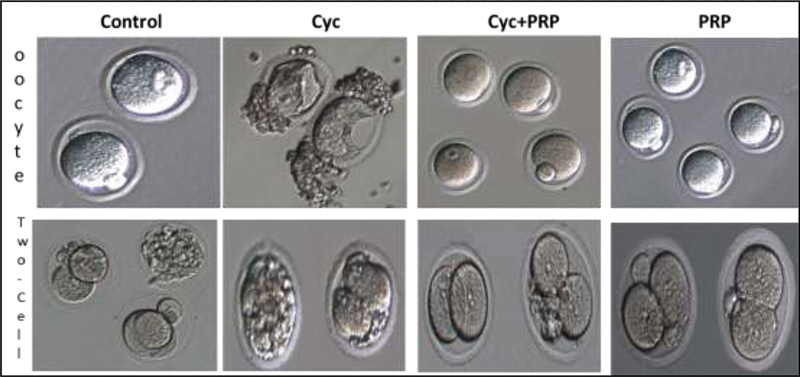
Oocyte and two-cell embryo images of control, CYC, CYC + PRP and PRP groups, 200X magnification in inverted microscope. There are two M2 oocytes belonging to the control group, 3 M2 and 1 GV oocyte in the CYC + PRP group and 4 M2 oocytes in the PRP group. In the CYC group, there are 2 GV oocytes whose ooplasms are severely damaged. In the CYC group, the quality of the 2-cell embryo is very poor, with a high rate of fragmentation. While two-cell embryos with equal blastomeres are seen in the CYC + PRP and PRP groups, embryos with a very small amount of fragmentation are seen in the CYC + PRP group. Although embryos with equal blastomeres were seen in the CYC + PRP and PRP groups, embryos with a small amount of fragmentation were seen in the CYC + PRP group. Abbreviations: Control, control group; CYC, cyclophosphamide administered group; CYC + PRP, cyclophosphamide and platelet-rich plasma applied group; PRP, platelet-rich plasma applied group; GV, germinal vesicle; MII, metaphase II.

Two-celled embryos were obtained by culturing oocytes after IVF. In the CYC group, the quality of the two-celled embryo was very poor, a high rate of fragmentation was seen. Although embryos with equal blastomeres were seen in the CYC + PRP and PRP groups, embryos with a small amount of fragmentation were seen in the CYC + PRP group. This effect was thought to be due to CYC. In the control group, embryos with equal blastomeres were generally seen, however, it was seen in embryos with fragmentation ( Fig. 3 ).

**Fig. 4:**
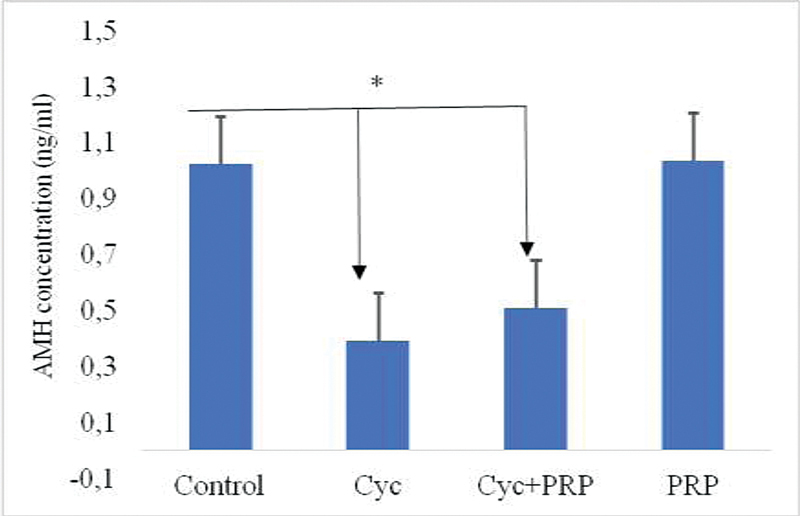
Comparison of experimental groups serum anti-Mullerian hormone (AMH) concentrations. Abbreviations: Control, control group; CYC, cyclophosphamide administered group; CYC + PRP, cyclophosphamide and PRP applied group; PRP, platelet-rich plasma applied group. *p* < 0.05, compared with the control group. Values are given as mean and standard error.

When the AMH concentrations in the study groups were examined, it was found that it was the highest in the PRP group, while it was the lowest in the CYC group ( Fig. 4 ). It was observed that there was a statistically significant difference between the CYC and CYC + PRP groups when compared with the control group ( *p* = 0.000).

## Discussion

For ovarian failure, the presence of ovarian atrophy, follicle reduction, and sex hormonal diminution are used. 23 Looking at society, ovarian failure (POI) is one of the most important diseases that cause infertility in women and threaten women's health. The early detection and treatment of ovarian dysfunctions continues to be an important research and clinical area of interest in gynecology. Infertile patients with aging ovaries - sometimes called the approaching POI, their numbers are increasing day by day and constitute a significant proportion of patients applying for IVF/ART. Current approaches to effective management of patients diagnosed with POI offer a wide range of options. Although egg donation (ED) is still the most successful and final treatment for POI patients, the vast majority of these infertile women are reluctant to consent to ED upon initial diagnostic interview and demand alternative solutions using their own autologous eggs, despite the low chance of success. 24 Many researchers have investigated the use of stem cell transplantation, including human menstrual blood stem cells, fat-derived stem cells, human endometrial mesenchymal stem cells, Platelet-rich plasma (PRP), as a cell therapy to reverse ovarian damage caused by chemotherapy. 12 16 25 26 PRP has been defined as a blood plasma fraction with a platelet concentration 4 to 5 times higher than the normal level, and its beneficial effect on tissue regeneration, angiogenesis activation, inflammation control and anabolism has already been demonstrated in many medical fields. 27 The main components of PRP that contribute to tissue healing and regeneration, anabolism increase, differentiation and proliferation, angiogenesis activation, inflammation control can be listed as hormones, macrophages, neutrophils, cytokines and various growth factors. 28 29 Therefore, the use of PRP is considered a justified and potentially successful opportunity to increase the fertility outcome in POI patients where the main problem is ovarian failure.

A POI model induced by cyclophosphamide (CYC) was used in the present study. Cyc is an alkylating agent which induce ovarian failure in animal models. 30 It has been shown in previous studies that CYC disrupts the ultrastructure of granulosa cells and induces apoptosis and autophagy and eventually causes ovarian failure. 30 31 Cyc has been shown to reduce ovarian weight and volume, reduce the number of different follicles and sex hormone levels, and increase atretic follicles. 12 In their study of agents that prevent chemotherapy-induced ovarian damage, Roness et al. 32 noted that AS-101, AMH, imatinib, sphingosine-1-phosphate, granulocyte colony stimulating factor, bortezomib, and multi-drug resistance gene-1 were effective in preventing chemotherapy-induced ovarian damage. 32 Different mechanisms of action associated with different protective agents have been shown to be effective, including inhibition of follicle activation, anti-apoptosis effects, vascular effects, and gene upregulation. 32 When this protective effect is evaluated in terms of PRP, there are studies showing the success of PRP. 12 16 These studies were generally performed on ovarian tissue and were performed on oocytes obtained at the stage of folliculogenesis.

Growth factors play an important role in improving the structure and function of the ovaries, and different growth factors such as VEGF, EGF, PDGF, and TGF-b have been shown to have protective effects on ovarian damage. 13 14 33 Platelet-rich plasma has a protective effect against ovarian damage caused by CYC, as it has high amounts of these factors in its structure. This efficiency has been demonstrated in previous studies. 12 16 This protective feature of PRP in POI patients is to protect follicle development and oocyte number during folliculogenesis. Except for the protective effects of PRP on the ovary, there are many studies on the effects of PRP on the endometrium. 34 It has been shown that intrauterine PRP treatment supports endometrial growth and improves assisted reproductive outcome in patients with thin endometrium. 35 In humans, PRP used in autologous ovarian transplantation to improve the vascularization and quality of the implant has been shown to increase transplant success resulting in live birth. 36 There is no study on the effectiveness of PRP for POI patients who have serious difficulties in IVF applications. Human studies on the subject in the literature are only at the level of case reports. 37 38

As a result of our study, it is seen that the addition of PRP treatment in the group where POI was created with CYC positively affected the results of subsequent IVF. This positive effect is valid for both the number of oocytes obtained by ovulation stimulation and the number of embryos on day 2 obtained after fertilization. When the day-2 embryos obtained were evaluated in terms of their quality, it was noted that there is a significant difference in the PRP applied group compared with the untreated group. This may be due to the fact that the PRP treatment could probably enrich the dysfunctional ovarian tissues with essential factors for neoangiogenesis, leading to tissue regeneration and reactivation. Although the effect of PRP on regenerative and repair processes in somatic tissues remains largely uncertain, growth factors contained in PRP content may have many critical roles in the ovaries through physiologically local effects such as cell growth, proliferation, differentiation, chemotaxis, angiogenesis, and formation. These growth factors control the release of the extracellular matrix and even other growth factors in close proximity to the release sites. 39 Platelet-rich plasma can accelerate this process while supporting the self-repair of ovaries, follicles after chemotherapy, which already have the potential to repair itself. 40

When the control group and the PRP-only group were compared, the number of oocytes obtained in the group receiving PRP and the number of embryos on day 2 were higher, but this result was not found to be statistically significant. This situation makes us think that PRP does not have a significant effect in conditions with normal ovarian function and reserve. It seems, the beneficial effect of PRP is only applied on damaged ovaries, and it has no effect on the normal structure for IVF cycles.

Anti-Mullerian hormone, a powerful marker of ovarian reserve, is a member of the transforming growth factor superfamily produced by the granulosa cells of the antral follicles in the ovary. 41 Considering the AMH levels, there was an increase in the PRP-CYC group compared with the CYC group. However, the AMH levels following PRP treatment corresponded to the expected lower AMH levels in a POI case, although an improvement in overall reproductive potential was observed. Although the PRP-CYC group had a low AMH level that could be diagnosed with POI compared with the control group, this decrease is not as extreme as in the group without PRP treatment, and it is not at a low level that will allow more oocytes and embryos to be obtained as a result of IVF. This suggested that PRP could improve ovarian reserve by protecting ovarian granulosa cells. This evidence demonstrated the protective effects of PRP from CYC damage to the ovarian follicles.

## Conclusion

The present study evaluated the number and quality of oocytes obtained after ovarian stimulation and the number and quality of embryos obtained on the second day after fertilization. Our study showed that PRP can protect the ovarian function against damage induced by CYC, but it provides an improvement in the number of oocytes and developing embryos as a result of the oocyte stimulation performed during the subsequent IVF procedure. However, investigating the implantation results of these embryos, and evaluating the ongoing pregnancy results will be a good target for future studies.
